# Occurrence and seasonal variation of organochlorine pesticides in selected vegetable farmlands in Lagos State, Nigeria

**DOI:** 10.5620/eaht.2024013

**Published:** 2024-04-15

**Authors:** Fidelia Ijeoma Osuala, Olamide Florence Humphrey, Miriam Nwana Igwo-Ezikpe, Arnold Godfrey Udoh, Iyanuoluwa Adegbuyi, Mojeed Fasasi, Precious Agada, Azeemah Jimoh, Olufunke Okubamowo

**Affiliations:** 1 Department of Zoology, Faculty of Science, University of Lagos, Lagos, Nigeria; 2 Department of Biochemistry, Faculty of Basic Medical Sciences, College of Medicine, University of Lagos, Lagos, Nigeria

**Keywords:** organochlorine pesticide residues, risk assessment, urban farming, agriculture zone, Lagos State

## Abstract

Pest infestation in crop production have increased farmers' interest in pesticides use with short and long term consequences. This study investigated the occurrence and seasonal variations of organochlorine pesticide residues in vegetable farms in selected areas of Lagos State. Non carcinogenic and carcinogenic risk assessment was also evaluated. Soil samples were collected during the wet and dry seasons at soil depth of 0-30 cm. Gas chromatography coupled with an Agilent mass spectrometer was used to analyse organochlorine residues (alpha-lindane,lindane, delta.-lindane, aldrin, heptachlor epoxide, alpha.-endosulfan, p,p'-dichlorodiphenyldichloroethylene (p,p'-DDE), endrin, endosulfan, m,p'-dichlorodiphenyldichloroethane (m,p'-DDD), endosulfan sulfate, o,p'-dichlorodiphenyltrichloroethane (o,p'-DDT) and endrin ketone) in soil. Heptachlor epoxide showed maximum concentration of 43.03 mg/kg in Station 19 in Western zone during the dry season while m,p'-DDD and endosulfan had minimum value of 0.004 mg/kg in Station 2 and Station 5 respectively during the wet season in the Far eastern zone. The concentrations of organochlorine residues were intermediate in the Eastern zone in both seasons. There was significant (p < 0.05) increase in dry season concentrations when compared to wet season. The risk assessment indicated Hazard Quotient (HQ) > 1 for non-cancer risk and cancer risk > 10-6. Thus a need for stringent monitoring programs for pesticides.

## Introduction

Agriculture is the foundation of the Nigerian economy and the main source of livelihood for the majority of the rural population [1], who, as farmers, produce food for consumption and raw materials for production and exportation [2]. However, migration of people from the rural areas has led to increased population in the urban areas [3]. In order to meet the huge demand on food due to increased urban population, urban farming has become essential.

Lagos State is divided into three agricultural zones in terms of the spatial distribution of urban farming communities by the Agricultural Development; these are Far eastern, Eastern and Western zones [4]. There are various farming activities in Lagos State but majority of crop farmers cultivate vegetables [5]. However, pest infestation has been observed to have significant impact on vegetable production, accounting for 20-60 % losses before harvest [6, 5]. Consequently, farmers rely heavily on pesticides to control these pests [7, 8].

Pesticides are used globally at a rate of approximately two million tonnes per year, with Europe accounting for 45 %, the United States for 25 %, and the rest of the world for 25 %, with Africa accounting for 5 %. They have been accredited with success in both agricultural and public health sectors [9]. However, their usage over the years have caused contamination of the environmental matrices such as air, soil, groundwater, surface water and undesirable residues accumulating in soil, soil organisms and food crops [10]. Therefore, posing a threat to human health and the ecosystem as a whole. The continuous use of organochlorine pesticides remains global issue due to their persistence and long-distance dispersal via ocean currents and atmospheric transport [11]. Resulting in their banned by the United Nations Stockholm Convention [12]. However, despite the prohibition, most of the noxious organochlorine compounds (OCPs) are still used both on agricultural crops and domestically in many underdeveloped countries [13].

Exposure to pesticides could occur through dietary route such as consumption of foods contaminated with pesticides and non-dietary route which are accidental ingestion of soil, inhalation of dust and dermal contact [14]. These exposures could result in both non-carcinogenic and carcinogenic effects such as endocrine system disruption, immune system suppression, neurological damage, cancer and death in children and adults [15, 16]. Some organochlorine pesticides such as dichlorodiphenyltrichloroethane (DDT) and its metabolites, aldrin, heptachlor and it metabolite are classified as probable human carcinogens [17]. OCPs exposure have been associated with brain, kidney, leukemia, lymphoma and breast cancers [18-20].

Studies have shown that organochlorine pesticide residues are widely distributed in Nigerian soil, water and cultivated food [21-26]. Conversely, there is dearth of information on organochlorine residues in Lagos State vegetable farmlands and non-dietary health risk assessments of these residues in children and adults. Hence, this research assessed some of the organochlorine residues in the agricultural soils to provide information that would be useful in environmental monitoring of pesticides in the soil. With a view of evaluating the health risk connected to the non- dietary exposure of these residues.

## Materials and Methods

### Study area

**Figure 1.**
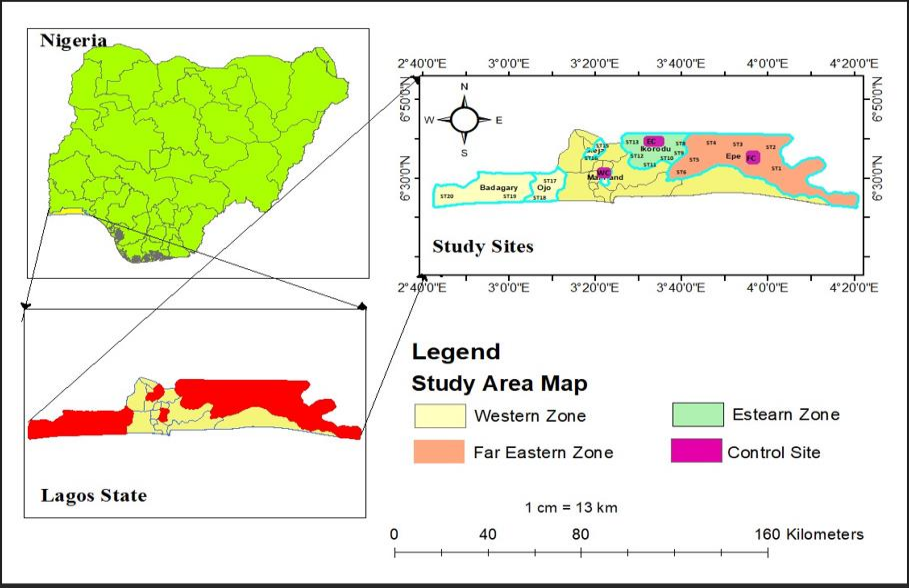
Map of the sampled stations

The study was carried out in selected areas in Lagos State with Latitude 6 ° 23′ N and 6 ° 41′ N, and Longitudes 3 ° 09′ E and 3 ° 20′. The sampling sites were selected based on the three zonal agricultural divisions of Lagos State by the Lagos State Agricultural Development Agency. From each zone, three blocks were selected where vegetables are cultivated. The Far eastern zone samples were collected from Epe (Origanringan), Ejirin, and Agbowa; were coded ST1-ST6; control site was designated as FC (ST7). The sampled sites in the Eastern zone were chosen from Imota, Gberigbe, and Laspotech; were coded ST8-ST13; control site was designated as EC (ST14). The Western zone sampled sites were chosen from Ikeja, Ojo and Badagry; were coded ST15 - ST20; control site was designated as WC (ST21). Six sampling locations were identified using the global positioning system and three sampling points were established for each location and zone. The control sites were locations within the zones where there were no obvious agricultural and industrial activities. A total of sixty three (63) soil samples were collected from different locations and the control sites. For each zone, seven (7) composite samples were analysed in triplicates for each season. The map of the study areas is shown in Figure 1.

### Sampling schedule and soil sample collection

Soil samples were collected between June/July and November/December, 2021 depicting the wet and dry seasons. Soil samples were collected using a clean stainless steel auger at a depth of 0 to 30 cm. Soil samples were placed in aluminum foil, kept in zip-lock bags in order to minimize possible contamination and stored at 4 °C as described by Opeolu et al. [27].

### Organochlorine pesticides residues extraction from Soil

Pesticides extraction from soil samples was performed using EPA 3550C method described by USEPA [28] with minor adjustment. The soil sample (10 g) was weighed into a 100 ml Erlenmeyer flask, 5 g of anhydrous sodium sulphate was added, followed by 50 ml of a mixture of acetone and n-hexane (1:1 v/v) to the mixture in the Erlenmeyer flask, sonicated for 10–15 minutes in a high-frequency ultrasound bath and decanted into a round bottom flask. The extraction process was repeated with another 50 ml (acetone and n-hexane). The extract was concentrated to 10 ml using a rotary evaporator.

### Extract clean-up process

A column was filled with cotton wool containing anhydrous silica gel and sodium sulphate (2:1). The column was conditioned with a mixture of 2.5 ml of n-hexane and acetone (1:1). The extract was then poured through the column and later eluted with 5 ml of the mixture of n-hexane and acetone. The eluate was concentrated to 2 ml using a nitrogen concentrator or by air-drying with exclusion of light. The eluate was poured into a GC vial for GC-MS analysis.

### Determination and quantification of Organochlorine pesticides in Soil samples

Gas chromatography (GC) was operated in selective ion monitoring (SIM) and scanning mode to ensure detection of target components at low concentration. The stationary phase for the separation of the compounds was an HP-5M capillary column (30 m length × 0.32 mm diameter × 0.25 m film thickness) coated with 5 % phenylmethylsiloxane (Agilent Technologies). The carrier gas was helium at a constant flow of 1.2 ml/min at an initial nominal pressure of 0.26 psi and an average velocity of 40.00 cm/sec. The samples were injected in splitless mode at an injection temperature of 250 °C. The flush flow used was 30.0 ml/min at 0.35 min with a total flow of 31.24 ml/min. The initial oven temperature was 50 °C (1 min) and then increased from 25 °C/min to 100 °C (3 mins) and from 5 °C/min to 300 °C (5 mins). The run time was 16 mins with a solvent delay of 3 mins. The mass spectrometer was set to electron impact ionization mode at 70 eV, with the ion source temperature set at 230 °C, the quadrupole temperature at 150 °C, and the transmission line temperature at 300 °C. Quantification of OCPs involved external calibration curves prepared from reference standard solution. The concentrations of organochlorine residues were determined by comparing the peak heights of the samples with the peak heights of the reference standards of known concentrations.

### Health risk assessment

Three possible routes of human exposure to pesticide residues in soil samples from the three Agricultural zones were considered in determining the risk to human health. The three exposure routes were ingestion, inhalation and dermal contact. This represents the non-dietary risk assessment.

### Chronic daily intake (CDI)

All routes of exposure were subjected to non-carcinogenic and carcinogenic risk assessments in children and adults using the risk assessment models [29, 30]. CDI of contaminated soil samples for each population (children and adults) were estimated using the equations 1 to 5 below [31,32] to evaluate the non-carcinogenic risk. Then, the carcinogenic risk for each population (children and adults) was estimated using the cancer slope factor (equation 6) [33]


(1) $$\mathrm{CDI}\,\mathrm{ingestion}=\frac{C_{\mathrm{soil}}\,\,\mathrm{InR}\,\mathrm{CF}\,\mathrm{ED}\,\mathrm{EF}}{\mathrm{AT}\,\mathrm{BW}}$$

(2) $$\mathrm{CDI}\,\mathrm{inhalation}=\frac{C_{\mathrm{soil}}\,\mathrm{InR}\,\mathrm{ED}\,\mathrm{EF}}{\mathrm{AT}\,\mathrm{BW}\,\mathrm{PEF}}$$

(3) $$\mathrm{CDI}\,\mathrm{dermal}=\frac{C_{\mathrm{soil}}\,\mathrm{CF}\,\mathrm{ED}\,\mathrm{EF}\,\mathrm{ABS}\,\mathrm{AF}\,\mathrm{SA}}{\mathrm{AT}\,\mathrm{BW}}$$

(4) $$\mathrm{HQ}=\frac{\mathrm{CDI}}{\mathrm{RfD}}$$

(5) HI = ∑ HQ

(6) CR = CDI × CSF

Where Csoil is the concentration organochlorine residue in soil (mg/kg); InR is the ingestion rate, rate (200 mg.d-1 for a child and 100 mg.d-1) CF is the conversion factor (106 mg/kg), ED is the exposure duration (yr), EF is the exposure frequency (350 d/yr for a child and adult), IhR is the inhalation rate (12 m^3^/d for child and 13.25 m^3^/d), AT is the average life span for (non-cancer risk: 2190 d for child and 9490 d for adult; cancer risk:25550 for both child and adult), BW is the average body weight (17.4 kg for a child and 80 kg for adult); PEF is the particle emission factor (1.36 x 10-9 m^3^/kg), ABS is the dermal absorption factor (chemical specific), AF is the skin adherence factor for soil (0.2 mg/cm^2^ for a child and 0.07 mg/cm^2^) for adult, SA is soil surface area (cm^2^), ABS is dermal absorption factor (chemical specific), RfD is reference oral dose (chemical specific),CDI: Chronic daily intake, HQ is Hazard Quotient, HI is Hazard Index, CR is Cancer Risk, CSF is Cancer Slope Factor

### Data analysis

The mean and standard deviations (mean±standard deviation) of obtained data on organochlorine and their residues in soil samples were computed. Two way ANOVA was carried out to compare the means and Duncan multiple range test was used to separate the means at level of significant (p < 0.05). The data were processed using the Software Package for Social Sciences (version 26.0).

## Results

### Far Eastern zone

Maximum concentration of Heptachlor epoxide (34.69 mg/kg) was detected in ST5 during the dry season while m,p'-DDD and endosulfan sulfate had the minimum concentration of 0.004 mg/kg in ST2 and ST5 respectively during the wet season. The concentrations of the OCP residues in the sampled stations during the wet and dry seasons varied significantly (p < 0.05) but there was no significant (p > 0.05) variation in OCP concentrations in some sampled stations and control site at both seasons (Table 1).

### Eastern zone

Heptachlor epoxide had 21.74 mg/kg had the highest concentration in ST12 during the wet season while m,p'- DDD and endosulfan sulfate had the lowest concentration of 0.004 mg/kg in ST8 and ST9 during the dry season. There was significant (p< 0.05) variation in some OCP residue concentrations across the sampled stations and control sites during the wet and dry seasons (Table 2).

### Western zone

In the western zone, heptachlor epoxide had maximum concentration of 43.03 mg/kg in ST19 during the dry season while alpha-endosulfan and endosulfan in ST15 and ST16 respectively with minimum concentration of 0.004 mg/kg in wet season. There was significant (p < 0.05) variation in the concentrations of some of the OCPs detected in the sampled stations when compared with control site during the wet and dry season (Table 3).

### Risk assessment

The results of the non-carcinogenic and carcinogenic risk of organochlorine pesticide residues in children and adults from the three agricultural zones are presented below.

### Far Eastern zone

Heptachlor epoxide had the highest HQ of 5.34 × 10^7^, 4.91 × 10^14^ and 1.76 while endosulfan sulfate had the lowest HQ with values of 1.89 × 10^2^, 1.73 × 10^9^ and 6.22 × 10^-6^ in children for ingestion, inhalation and dermal exposure routes respectively. Likewise, heptachlor epoxide had the highest HQ in adults with values of 1.21, 1.18 × 10^14^ and 4.61 × 10^7^ while endosulfan sulfate had the lowest HQ with values of 4.28 × 10^-6^, 4.17 × 10^8^ and 1.63 × 10^2^ for the three exposure routes respectively. HQ for other organochlorine residues had intermediate values. All the organochlorine residues had HI > 1 for all exposure routes in children and adults (Table 4). The total cancer risks in children for inhalation and ingestion routes was significantly greater than the stipulated potential cancer risk values of 10^-1^ while the dermal route in children had cancer risk value within the stipulated values of 10^-6^ and 10^-4^. Adults had cancer risk value within the stipulated values of 10^-6^ and 10^-4^ for inhalation and dermal routes while the inhalation route had value significantly greater than the stipulated potential cancer risk values of 10^-1^ (Table 4).

### Eastern zone

Heptachlor epoxide had the highest HQ of 4.31 × 10^6^, 3.96 × 10^13^ and 1.42 × 10^-1^ for ingestion, inhalation and dermal exposure routes respectively while p,p'-DDE had the lowest HQ with values 5.29 × 10^1^, 4.86 × 10^8^ and 1.74 × 10^-6^ for ingestion, inhalation and dermal exposure routes respectively in children. Also, heptachlor epoxide had the highest HQ in adults with values of 9.76 × 10^-2^, 9.51 × 10^12^ and 3.71 × 10^6^ for ingestion and inhalation and dermal routes respectively while p,p'-DDE had the lowest HQ with values of 1.2 × 10^-6^ and 1.17 × 10^8^; 4.56 × 10^1^ for ingestion, inhalation and dermal exposure routes respectively. HQ for other organochlorine residues had intermediate values. All the organochlorine residues had HI < 1 for dermal exposure in children while adults had HI < 1 for ingestion route (Table 5). The total cancer risks in children and adults for ingestion and dermal exposure routes were within the stipulated potential cancer risk values (10^-6^ to 10^-1^) while children and adults had values greater than the stipulated value (10^-1^) for inhalation exposure route (Table 5).

### Western zone

Heptachlor epoxide had the highest HQ of 3.57 × 10^7^, 3.28 × 10^14^ and 1.18 while endosulfan sulfate had lowest HQ of 1.11 × 10^2^, 1.02 × 10^9^ and 3.66 × 10^-6^ for ingestion, inhalation and dermal exposure routes respectively in children. In adults, heptachlor epoxide had the highest HQ of 8.09 × 10^-1^, 7.89 × 10^13^ and 3.08 × 10^7^ while endosulfan sulfate had lowest HQ of 2.52 × 10^-6^, 2.45 × 10^8^ and 9.57 × 10^1^ for ingestion, inhalation and dermal exposure routes respectively. HQ for other organochlorine residues had intermediate values. All the organochlorine residues had HI > 1 for all exposure routes in children. Adults had HI < 1 in ingestion route while inhalation and dermal routes had HI > 1 (Table 6). The total cancer risk values in children and adults were greater than the stipulated potential cancer risk values (10-1) for inhalation exposure route, (< 10^-4^) for ingestion and dermal exposure routes respectively (Table 6).

## Discussion

Pesticides especially organochlorine pesticides are extremely toxic, less than 1 % hit the target, when applied [34]. The remaining pesticides lingers in the soil for a long time before breaking down into metabolites, which can be dangerous depending on their chemical composition [35-37]. Several studies have shown pesticide residues in different environmental matrices and organisms [26, 38-39]. These concentrations showed the indiscriminate and injurious use of these pesticides in Nigeria regardless of government policy on their usage [40].

Heptachlor epoxide which was the most prominent OCP residue detected in this study is the degradation product of heptachlor has low susceptibility to biodegradation, oxidation and hydrolysis or photolysis in the environment [41]. Serious heptachlor epoxide poisoning could cause central nervous system effects as such seizures, convulsions and hyperexcitability, liver damage and cancer [42-43]. The high concentration of heptachlor epoxide in this study could be as a result of recent application or accumulation of its massive usage for a long period of time in the past. The value obtained was higher than the findings of Gopalan and Chenicherry (2018) [44] who detected 7.5 ng/g of heptachlor epoxide in soil during the monsoon season. Also, Huang et al. [33] detected lower concentration of heptachlor epoxide (0.51 ng/g) in their study.

Pollution from organochlorine pesticides occurs more frequently through transport and atmospheric deposition, which are responsible for their transport to distant locations [45]. The detection of these pesticide residues in the control sites in this study could be attributed to their trans boundary properties and this suggests drift during pesticide application, this is in agreement with the study of Tariq et al. [46].

In Nigeria clime, seasonality is differentiated by rainfall pattern [47]. Due to high intensity of rain during the wet season, these pollutants could be easily leached to the aquifer or runoff to the surrounding water bodies. Thereby, reducing their concentrations in wet season compared to the dry season. Seasonal variation, which was observed in the concentrations of OCP residues in this study could be attributed to the rainfall pattern. In addition, it also suggested their intensive use during the dry. This agrees with the study of Nyantaky et al. [48].

The Stockholm Convention identified and classified some of the organochlorine pesticides as probable carcinogens to human [49-51]. This shows that they could be detrimental to human and the environment. Non-carcinogenic health effects are shown by hazard index. HI > 1 specifies high potential non-carcinogenic health effects of such pesticide while HI < 1 indicates low non-carcinogenic health risk of the pesticide to human [52, 53]. ATSDR (1996) and (2004) [54, 55] standard proposed the following qualitative ranking of cancer risk: very low (value < 10^−6^), low (10^−6^ ≤ value < 10^−4^), moderate (10^−4^ ≤ value < 10^−3^), high (10^−3^ ≤ value < 10^−1^), and very high (value ≥ 10^−1^). The non-carcinogenic and carcinogenic risk assessment of organochlorine pesticide residues showed that most of the children and adults exposed to pesticides in the study areas could be at non-cancer and cancer health risk at different levels. This contradict the findings of Yao et al. [56], who observed that OCPs could have no non-carcinogenic risk to children but have some non-carcinogenic risk to adults. Their findings also showed that there was no carcinogenic risk when children and adults are exposed to OCPs through the three exposure routes.

## Conclusions

These results established that some of the vegetable farmlands in selected areas of Lagos State are polluted with organochlorine pesticides. This is an indication that these banned pesticide though they persist in the environment are still in use. Consequently, this could constitutes a threat to public health because of their potential bioaccumulation and biomagnification along the food chain when the cultivated vegetables are consumed. Therefore, there is need for more stringent monitoring program such as restriction on pesticide usage. This will prevent illegal use of banned pesticides and limit organisms’ exposure.

## Figures and Tables

**Table 1.** Wet and dry season’s organochlorine distribution (mg/kg) in the Far eastern zone

Target compounds	ST1		ST2		ST3		ST4		ST5		ST6		FC	
	WET	DRY	WET	DRY	WET	DRY	WET	DRY	WET	DRY	WET	DRY	WET	DRY
alpha.-Lindane	-	0.059±0.07	-	0.075±0.09	-	-	-	0.28±0.14	-	0.08±0.09	-	-	-	-
Lindane	0.029±0.001^a^	0.005±0.005^b^	0.019±0.001^b^	0.01±0.001^a^^b^	-	0.01±0.002^a^^b^	0.006±0.007^c^	0.015±0.006^a^^b^	-	0.009±0.002^a^^b^	-	0.034±0.028^a^	-	0.025±0.03^a^^b^
delta.-Lindane	0.104±0.028^a^	0.019±0.01^c^	0.044±0.034^b^	0.07±0.016^a^	0.029±0.0^b^^c^	0.019±0.002^c^	0.014±0.006^b^^c^	0.07±0.008^a^^b^	0.005±0.005^c^	0.039±0.02^c^	-	0.043±0.02^b^^c^	0.010±0.0003^b^^c^	0.034±0.03^c^
Aldrin	0.159±0.127^a^	0.02±0.0005^c^	0.084±0.064^a^^b^	0.12±0.04^a^^b^	0.030±0.0002^b^	0.073±0.05^b^^c^	0.039±0.023^b^	0.153±0.03^a^	0.020±0.0007^b^	0.073±0.06^b^^c^	0.008±0.002^b^	0.138±0.06^a^^b^	0.025±0.03^b^	0.174±0.017^a^
Heptachlor epoxide	0.085±0.07^c^	0.049±0.023^c^	0.044±0.019^c^	33.61±2.42^a^	0.029±0.002^c^	1.96±0.68^c^	12.70±1.42^b^	29.96±9.73^a^	21.37±1.29^a^	34.69±1.20^a^	0.029±0.001^c^	32.70±1.35^a^	-	13.45±0.96^b^
alpha.-Endosulfan	0.057±0.044^a^	0.009±0.002^b^	0.039±0.011^a^^b^	0.574±0.16^a^^b^	-	0.26±0.09^a^^b^	0.015±0.017^b^^c^	0.65±0.47^a^^b^	0.019±0.011^b^^c^	0.88±0.21^a^	-	0.36±0.40^a^^b^	0.02±0.0002^b^^c^	0.83±0.88^a^
p,p'-DDE	0.089±0.103^a^	-	-	0.009±0.01^a^	-	0.005±0.005^a^	0.005±0.006^b^	0.005±0.005^a^	-	-	-	0.01±0.01^a^	-	0.005±0.005^a^
Endrin	0.199±0.07^a^	0.093±0.006^c^	0.162±0.04^b^^c^	0.383±0.167^a^^b^	-	0.139±0.03^c^	0.300±0.07^c^^d^	0.49±0.27^a^	0.045±0.005^d^^e^	0.57±0.14^a^	0.029±0.001^e^	0.36±0.14^a^^b^	0.034±0.007^d^^e^	0.18±0.06^b^^c^
Endosulfan	0.039±0.012^a^	0.024±0.02^b^	0.040±0.011^a^	0.15±0.03^a^	-	0.024±0.007^b^	0.026±0.03^a^^b^	0.089±0.008^b^	0.039±0.023^a^	0.04±0.02^b^	-	0.074±0.05^b^	-	0.058±0.02^b^
m,p'-DDD	0.014±0.005^a^	-	0.004±0.004^b^	0.01±0.001^a^	-	-	-	0.009±0.002^a^^b^	-	0.009±0.001^a^	-	0.005±0.0005^b^^c^	-	0.004±0.005^b^^c^
Endosulfan sulfate	0.024±0.006^a^^b^	0.005±0.006^c^	0.009±0.01^b^	0.094±0.03^a^	-	0.008±0.002^c^	0.035±0.04^a^	0.024±0.006^b^^c^	0.004±0.005^b^	0.019±0.001^b^^c^	-	0.044±0.03^b^	-	0.029±0.01^b^^c^
o,p'-DDT	0.052±0.039^a^	-	0.009±0.002^b^	0.009±0.001^b^	-	0.009±0.001^b^	0.005±0.005^b^	0.035±0.006^a^	0.004±0.005^b^	0.029±0.01^a^	-	0.009±0.002^b^	-	0.004±0.005^b^^c^
Endrin ketone	0.228±0.127^b^	0.084±0.06^b^^c^	0.313±0.05^a^	0.245±0.21^a^^b^	-	0.068±0.01^c^	0.103±0.01^c^	0.263±0.09^a^	0.030±0.03^c^	0.23±0.10^a^^b^	0.019±0.001^c^	0.11±0.06^a^^b^^c^	0.028±0.038^c^	0.09±0.03^b^^c^

ST1 to ST6= sampling stations; FC= Control site for the Far eastern zone; Means are presented with ± standard deviation; - stands not detected. Mean values with different alphabets superscripts varied significantly (P < 0.05) across the stations

**Table 2.** Wet and dry season’s organochlorine distribution (mg/kg) in the Eastern zone

Target compounds	ST8		ST9		ST10		ST11		ST12		ST13		EC	
	WET	DRY	WET	DRY	WET	DRY	WET	DRY	WET	DRY	WET	DRY	WET	DRY
alpha.-Lindane	-	-	-	-	-	-	-	-	-	0.009±0.001^a^	-	-	0.029±0.033^a^	-
Lindane	0.030±0.001^c^	0.01±0.01^a^	0.044±0.006^b^	-	0.005±0.005^d^	-	0.005±0.005^d^	-	0.004±0.005^d^	-	-	-	0.054±0.006^a^	-
delta.-Lindane	0.114±0.041^a^	0.028±0.01^a^	0.029±0.002^b^	0.009±0.01^b^	0.009±0.0015^b^	-	0.022±0.032^b^	-	0.008±0.002^b^	-	0.009±0.001^b^	-	0.029±0.001^b^	-
Aldrin	0.054±0.006^a^^b^	0.024±0.006^a^^b^^c^	0.029±0.011^b^	0.03±0.02^a^^b^	0.01±0.0005^b^	0.018±0.01^a^^b^^c^	0.02±0.01^b^	0.014±0.005^b^^c^^d^	0.025±0.005^b^	-	0.025±0.006^b^	0.009±0.001^c^^d^	0.103±0.10^a^	0.034±0.006^a^
Heptachlor epoxide	0.119±0.036^c^	10.26±1.39^a^	0.080±0.01^c^	1.83±0.31^b^	0.019±0.001^c^	0.03±0.02^c^	3.23±0.62^b^	-	21.74±1.87^a^	-	0.023±0.009^c^	0.019±0.001^c^	0.135±0.066^c^	0.054±0.006^c^
alpha.-Endosulfan	0.064±0.051^a^	0.029±0.02^a^	0.0089±0.001^b^	-	-	-	0.004±0.005^b^	-	0.024±0.006^b^	-	-	-	0.008±0.002^b^	-
p,p'-DDE	-	0.004±0.005^a^	-	-	-	-	-	-	-	-	-	-	-	-
Endrin	0.099±0.046^b^^c^	0.216±0.03^a^	0.178±0.034^b^	0.093±0.04^b^	0.033±0.007^c^	0.024±0.005^c^	0.039±0.024^c^	0.019±0.001^c^	0.103±0.017^b^^c^	0.015±0.006^c^	0.079±0.034^b^^c^	0.009±0.001^c^	0.318±0.20^a^	0.018±0.002^c^
Endosulfan	0.059±0.011^a^	0.029±0.02^a^	0.028±0.01^b^	0.014±0.006^b^	-	-	0.015±0.005^c^^d^	-	0.014±0.007^c^^d^	-	0.004±0.005^d^^e^	-	0.024±0.006^b^^c^	-
m,p'-DDD	0.019±0.002^a^	0.004±0.005^a^	0.014±0.005^b^	-	-	-	-	-	-	-	-	-	0.004±0.005^c^	-
Endosulfan sulfate	0.014±0.016^a^^b^	0.014±0.006^a^	0.014±0.016^a^^b^	0.004±0.005^b^	-	-	0.004±0.005^b^	-	0.005±0.006^a^^b^	-	0.020±0.022^a^^b^	-	0.025±0.005^a^	-
o,p'-DDT	0.01±0.011^a^	0.005±0.005^a^	-	-	-	-	-	-	0.005±0.005^a^^b^	-	-	-	0.005±0.006^a^^b^	-
Endrin ketone	0.193±0.168^a^	0.18±0.11^a^	0.164±0.05^a^	0.049±0.02^b^	0.033±0.018^a^^b^	0.016±0.003^b^	0.059±0.01^c^	0.024±0.005^b^	0.06±0.0007^b^^c^	0.024±0.006^b^	0.039±0.024^c^	0.014±0.006^b^	0.153±0.006^a^^b^	0.024±0.007^b^

ST8 to ST13= sampling stations; EC= Control site for the Eastern zone; Means are presented with ± standard deviation; - stands not detected. Mean values with different alphabets superscripts varied significantly (P < 0.05) across the stations

**Table 3.** Wet and dry season’s organochlorine distribution (mg/kg) in the Western zone

Target compounds	ST15		ST16		ST17		ST18		ST19		ST20		WC	
	WET	DRY	WET	DRY	WET	DRY	WET	DRY	WET	DRY	WET	DRY	WET	DRY
alpha.-Lindane	-	-	-	0.26±0.02^a^	-	-	-	-	-	-	-	-	-	-
Lindane	0.005±0.005^b^	0.009±0.001^a^^b^	0.005±0.005^b^	0.008±0.001^a^^b^	0.005±0.005^b^	0.018±0.003^a^^b^	0.019±0.031^a^^b^	-	0.019±0.001^a^^b^	0.026±0.03^a^	0.020±0.023^a^^b^	0.014±0.005^a^^b^	0.044±0.019^a^	0.009±0.001^a^^b^
delta.-Lindane	-	0.034±0.029^a^^b^	0.014±0.005^b^	0.048±0.01^a^	0.008±0.002^b^	0.034±0.006^a^^b^	0.035±0.040^b^	-	0.036±0.004^b^	0.025±0.007^b^^c^	0.018±0.011^b^	0.025±0.005^b^^c^	0.089±0.07^a^	0.009±0.0014^c^^d^
Aldrin	0.040±0.011^b^	0.17±0.016^a^	0.024±0.006^b^	0.103±0.03^b^	0.014±0.006^b^	0.06±0.001^b^^c^	0.013±0.007^b^	-	0.024±0.006^b^	0.059±0.008^b^	0.034±0.007^b^^c^	0.089±0.03^b^	0.124±0.08^a^	0.09±0.08^b^
Heptachlor epoxide	2.39±0.34^b^	12.82±1.03^d^	11.61±0.16^a^	30.89±0.73^b^	0.020±0.001^c^	0.75±0.12^e^	0.015±0.017^c^	-	0.054±0.006^c^	43.03±0.13^a^	0.059±0.034^c^	0.44±0.08^e^	0.139±0.059^c^	20.67±0.52^c^
alpha.-Endosulfan	0.004±0.005^b^	0.58±0.59^b^^c^	0.008±0.002^b^	1.06±0.005^a^	-	0.418±0.013^c^	0.014±0.006^b^	-	0.029±0.022^b^	0.833±0.07^a^^b^	0.005±0.005^a^^b^	0.308±0.15^c^^d^	0.059±0.057^a^	0.043±0.03^d^
p,p'-DDE	-	0.005±0.005^b^	-	-	-	0.009±0.002^a^	0.005±0.006^a^	-	-	-	-	0.007±0.004^a^^b^	-	-
Endrin	0.074±0.06^b^	0.192±0.005^c^^d^^e^	0.139±0.12^a^^b^	0.45±0.32^b^	0.039±0.011^b^	0.369±0.01^b^^c^	0.070±0.03^b^	0.048±0.01^e^	0.202±0.01^a^^b^	0.65±0.02^a^	0.149±0.07^a^^b^	0.268±0.12^b^^c^^d^	0.272±0.25^a^	0.124±0.006^d^^e^
Endosulfan	0.019±0.001^b^	0.06±0.02^a^	0.004±0.005^b^	0.028±0.01^b^	0.019±0.032^b^	0.018±0.001^b^	0.005±0.005^b^	-	0.047±0.009^a^	0.06±0.001^a^	0.023±0.018^a^^b^	0.019±0.002^b^	0.046±0.021^a^^b^	0.024±0.02^b^
m,p'-DDD	-	0.005±0.006^a^^b^	-	0.009±0.001^a^	-	0.009±0.002^a^	-	-	0.014±0.006^a^	0.009±0.002^a^	0.009±0.01^a^^b^	0.004±0.005^a^^b^	0.01±0.01^a^^b^	-
Endosulfan sulfate	0.010±0.0006^a^	0.019±0.02^a^	0.029±0.033^a^	0.019±0.002^a^	0.019±0.022^a^	0.019±0.001^a^	0.005±0.005^a^	-	0.009±0.01^a^	0.019±0.001^a^	-	0.014±0.005^a^^b^	0.01±0.01^a^	0.009±0.01^a^^b^
o,p'-DDT	-	0.005±0.006^d^	-	0.029±0.01^a^^b^	-	0.029±0.001^a^	-	-	0.005±0,005^a^	0.018±0.002^c^	-	0.019±0.01^b^^c^	0.005±0.005^a^	-
Endrin ketone	0.089±0.07^b^^c^	0.224±0.10^a^^b^	0.104±0.018^b^	0.24±0.14^a^	0.055±0.006^b^^c^	0.14±0.005^a^^b^^c^	0.019±0.012^c^	0.019±0.001^d^	0.194±0.09^a^	0.22±0.02^a^^b^	0.093±0.03^b^^c^	0.119±0.05^b^^c^^d^	0.105±0.06^b^	0.01±0.04^c^^d^

ST15 to ST20= sampling stations; WC= Control site for the Western zone; Means are presented with ± standard deviation; - stands not detected. Mean values with different alphabets superscripts varied significantly (P < 0.05) across the stations

**Table 4.** Estimation of non-carcinogenic and carcinogenic for organochlorine residues in Far eastern zone

OCPs	Non-carcinogenic							Carcinogenic					
	Children			Adults				Children			Adults		
	HQ_ingestion_	HQ_inhalation_	HQ_dermal_	HQ_ingestion_	HQ_inhalation_	HQ_dermal_		CR_ingestion_	CR_inhalation_	CR_dermal_	CR_ingestion_	CR_inhalation_	CR_dermal_
alpha.-Lindane	5.90×10^3^	5.43×10^10^	1.95×10^-4^	1.34×10^-4^	1.3×10^10^	5.09×10^3^		1.99×10^-7^	-	5.17×10^-8^	9.4×10^-8^	-	3.97×10^-8^
Lindane	2.04×10^3^	1.88×10^10^	2.7×10^-5^	4.63×10^-5^	4.52×10^9^	7.05×10^2^		1.42×10^-8^	1.45×10^-1^	1.48×10^-9^	6.71×10^-9^	1.51×10^-1^	1.13×10^-9^
delta.-Lindane	6.36×10^3^	5.85×10^10^	2.1×10^-4^	1.44×10^-4^	1.41×10^10^	5.48×10^3^		6.14×10^-8^	-	1.59×10^-8^	2.89×10^-8^	-	1.22×10^-8^
Aldrin	1.43×10^5^	1.31×10^12^	4.×10^-3^	3.23×10^-3^	3.15×10^11^	1.23×10^5^		1.3×10^-6^	1.65×10^1^	3.37×10^-7^	6.12×10^-7^	1.72×10^1^	2.58×10^-7^
Heptachlor epoxide	5.34×10^7^	4.91×10^14^	1.76	1.21	1.18×10^14^	4.61×10^7^		1.13×10^-4^	1.42×10^3^	2.93×10^-5^	5.32×10^-5^	1.48×10^3^	2.25E-05
alpha.-Endosulfan	2.37×10^3^	2.18×10^10^	7.83×10^-5^	5.38×10^-5^	5.25×10^9^	2.05×10^3^		-	-	-	-	-	-
p,p'-DDE	1.62×10^3^	1.49×10^10^	5.34×10^-5^	3.68×10^-5^	3.58×10^9^	1.40×10^3^		2.96×10^-9^	3.72×10^-2^	7.66×10^-10^	1.39×10^-9^	3.87×10^-2^	5.88×10^-10^
Endrin	3.93×10^4^	3.61×10^11^	1.30×10^-3^	8.91×10^-4^	8.68×10^10^	3.39×10^4^		-	-	-	-	-	-
Endosulfan	3.90×10^2^	3.59×10^9^	1.29×10^-5^	8.85×10^-6^	8.62×10^8^	3.36×10^2^		-	-	-	-	-	-
m,p'-DDD	6.87×10^3^	6.32×10^10^	2.27×10^-5^	1.56×10^4^	1.52×10^10^	5.92×10^3^		-	-	-	-	-	-
Endosulfan sulfate	1.89×10^2^	1.73×10^9^	6.22×10^-6^	4.28×10^-6^	4.17×10^8^	1.63×10^2^		-	-	-	-	-	-
o,p'-DDT	1.27×10^3^	1.17×10^10^	4.18×10^-5^	2.88×10^-5^	2.8×10^9^	1.09×10^3^		-	-	-	-	-	-
Endrin ketone	-	-	-	-	-	-		-	-	-	-	-	-
HI	5.36×10^7^	4.93×10^14^	1.77	1.22	1.18×10^14^	4.62×10^7^	TCR	1.15×10^4^	1.44×10^3^	2.97×10^-5^	5.4×10^-5^	1.50×10^3^	2.28×10^-5^

HQ stands for Hazard quotient, HI: Hazard index, CR: Cancer risk, TCR stands for Total cancer risk

**Table 5.** Estimation of non-carcinogenic and carcinogenic for Organochlorine residues in Eastern zone

OCPs	Non-carcinogenic							Carcinogenic					
	Children			Adults				Children			Adults		
	HQ_ingestion_	HQ_inhalation_	HQ_dermal_	HQ_ingestion_	HQ_inhalation_	HQ_dermal_		CR_ingestion_	CR_inhalation_	CR_dermal_	CR_ingestion_	CR_inhalation_	CR_dermal_
alpha.-Lindane	4.29×10^2^	3.89×10^9^	1.39×10^-5^	9.59×10^-6^	9.34×10^8^	3.65×10^2^		1.43×10^-8^	-	3.7×10^-9^	6.73×10^-9^	-	2.84×10^-9^
Lindane	3.01×10^3^	2.77×10^10^	3.97×10^-5^	6.83×10^-5^	6.66×10^9^	1.04×10^3^		2.×10^-8^	2.14×10^-1^	2.18×10^-9^	9.9×10^-9^	2.23×10^-1^	1.67×10^-9^
delta.-Lindane	4.21×10^3^	3.87×10^10^	1.39×10^-4^	9.55×10^-5^	9.3×10^9^	3.63×10^3^		4.06×10^-8^	-	1.05×10^-8^	1.92×10^-8^	-	8.08×10^-9^
Aldrin	5.44×10^4^	5.01×10^11^	1.80×10^-3^	1.2×10^-3^	1.2×10^11^	4.69×10^4^		4.96×10^-7^	6.31	1.29×10^-7^	2.34×10^-7^	6.567378	9.87×10^-8^
Heptachlor epoxide	4.31×10^6^	3.96×10^13^	1.42×10^-1^	9.76×10^-2^	9.51×10^12^	3.71×10^6^		9.1×10^-6^	1.15×10^2^	2.36×10^-6^	4.29×10^-6^	119.3831	1.81×10^-6^
alpha.-Endosulfan	1.06×10^2^	9.73×10^8^	3.49×10^-6^	2.4×10^-6^	2.34×10^8^	9.11×10^1^		-	-	-	-	-	-
p,p'-DDE	5.29×10^1^	4.86×10^8^	1.74×10^-6^	1.2×10^-6^	1.17×10^8^	4.56×10^1^		9.64×10^-11^	1.21×10^-3^	2.5×10^-11^	4.54×10^-11^	1.26×10^-3^	1.92×10^-11^
Endrin	1.66×10^4^	1.53×10^11^	5.47×10^-4^	3.76×10^-4^	3.66×10^10^	1.43×10^4^		-	-	-	-	-	-
Endosulfan	1.25×10^2^	1.15×10^9^	4.12×10^-6^	2.84×10^-6^	2.76×10^8^	1.08×10^2^		-	-	-	-	-	-
m,p'-DDD	5.11×10^3^	4.7×10^10^	1.68×10^-4^	1.16×10^-4^	1.13×10^10^	4.40×10^3^		-	-	-	-	-	-
Endosulfan sulfate	7.49×10^1^	6.89×10^8^	2.47×10^-6^	1.7×10^-6^	1.65×10^8^	6.46×10^1^		-	-	-	-	-	-
o,p'-DDT	1.80×10^2^	1.65×10^9^	5.93×10^-6^	4.08×10^-6^	3.97×10^8^	1.55×10^2^		-	-	-	-	-	-
Endrin ketone	-	-	-	-	-	-		-	-	-	-	-	-
HI	4.39×10^6^	4.04×10^13^	1.45×10^-1^	9.95×10^-2^	9.7×10^12^	3.78×10^6^	TCR	9.68×10^-6^	1.21×10^2^	2.51×10^-6^	4.56×10^-6^	1.26×10^2^	1.92×10^-6^

HQ stands for Hazard quotient, HI: Hazard index, CR: Cancer risk, TCR stands for Total cancer risk

**Table 6.** Estimation of non-carcinogenic and carcinogenic for Organochlorine residues in Western zone

OCPs	Non-carcinogenic							Carcinogenic					
	Children			Adults				Children			Adults		
	HQ_ingestion_	HQ_inhalation_	HQ_dermal_	HQ_ingestion_	HQ_inhalation_	HQ_dermal_		CR_ingestion_	CR_inhalation_	CR_dermal_	CR_ingestion_	CR_inhalation_	CR_dermal_
alpha.-Lindane	3.26×10^3^	3×10^10^	1.07×10^-4^	7.39×10^-5^	7.2×10^9^	2.81×10^3^		1.1×10^-7^	-	2.85×10^-8^	5.19×10^-8^	-	2.19×10^-8^
Lindane	2.50×10^3^	2.3×10^10^	3.3×10^-5^	5.67×10^-5^	5.53×10^9^	8.63×10^2^		1.74×10^-8^	1.78×10^-1^	1.81×10^-9^	8.22E-09	1.85×10^-1^	1.39×10^-9^
delta.-Lindane	4.74×10^3^	4.36×10^10^	1.56×10^-4^	1.07×10^-4^	1.05×10^10^	4.09×10^3^		4.57×10^-8^	-	1.19×10^-8^	2.16×10^-8^	-	9.1×10^-9^
Aldrin	1.09×10^5^	9.98×10^11^	3.58×10^-3^	2.46×10^-3^	2.4×10^11^	9.36×10^4^		9.89×10^-7^	1.26×10^1^	2.56×10^-7^	4.66×10^-7^	1.31×10^1^	1.97×10^-7^
Heptachlor epoxide	3.57×10^7^	3.28×10^14^	1.18	8.09×10^-1^	7.89×10^13^	3.08×10^7^		7.55×10^-5^	9.51×10^2^	1.96×10^-5^	3.56×10^-5^	9.90×10^2^	1.5×10^-5^
alpha.-Endosulfan	2.12×10^3^	1.95×10^10^	6.98×10^-5^	4.8×10^-5^	4.68×10^9^	1.83×10^3^		-	-	-	-	-	-
p,p'-DDE	3.17×10^2^	2.92×10^9^	1.05×10^-5^	7.19×10^-6^	7.01×10^8^	2.73×10^2^		5.78×10^-10^	7.28×10^-3^	1.5×10^-10^	2.72×10^-10^	7.57×10^-3^	1.15×10^-10^
Endrin	3.84×10^4^	3.53×10^11^	1.27×10^-2^	8.7×10^-4^	8.47×10^10^	3.31×10^4^		-	-	-	-	-	-
Endosulfan	2.33×10^2^	2.14×10^9^	7.67×10^-6^	5.27×10^-6^	5.14×10^8^	2.01×10^2^		-	-	-	-	-	-
m,p'-DDD	8.46×10^3^	7.78×10^10^	2.79×10^-4^	1.92×10^-4^	1.87×10^10^	7.29×10^3^		-	-	-	-	-	-
Endosulfan sulfate	1.11×10^2^	1.02×10^9^	3.66×10^-6^	2.52×10^-6^	2.45×10^8^	9.57×10^1^		-	-	-	-	-	-
o,p'-DDT	8.25×10^2^	7.59×10^9^	2.72×10^-5^	1.87×10^-5^	1.82×10^9^	7.11×10^2^		-	-	-	-	-	-
Endrin ketone	-	-	-	-	-	-		-	-	-	-	-	-
HI	3.59×10^7^	3.3×10^14^	1.18	8.13×10^-1^	7.92×10^13^	3.09×10^7^	TCR	7.66×10^-5^	9.64×10^2^	1.99×10^-5^	3.61×10^-5^	1.00×10^3^	1.52×10^-5^

HQ stands for Hazard quotient, HI: Hazard index, CR: Cancer risk, TCR stands for Total cancer risk

## References

[1] Food and Agriculture Organization of the United Nations (2019). FAO source framework - Migration as a choice and an opportunity for rural development. FAO.

[2] Adeniyi DA, Dinbabo MF (2020). Efficiency, food security and differentiation in small-scale irrigation agriculture: Evidence from North West Nigeria. Cogent Social Sciences.

[3] Agwu AE, Anugwa IQ, Ifeonu CF (2021). Stemming rural-urban migration through agricultural development: Can Nigeria apply the lessons from the COVID-19 pandemic?. Agro-Science.

[4] Odudu CO (2015). An examination of tenure security for urban crop farming in Lagos, Nigeria. Ethiopian Journal of Environmental Studies and Management.

[5] Ofuya TI, Okunlola AI, Mbata GN (2023). A review of insect pest management in vegetable crop production in Nigeria. Insects.

[6] Okunlola AI, Ofuya TI, Aladesanwa RD (2008). Efficacy of plant extracts on major insect pests of selected leaf vegetables in Southwestern Nigeria. Agricultural Journal.

[7] Denloye AA, Makinde OSC, Ajelara KO, Alafia AO, Oiku EA, Dosumu OA (2014). Insects infesting selected vegetables in Lagos and the control of infestation on Celosia Argentea (L.) with two plant essential oils. International Journal of Pure and Applied Zoology.

[8] Philbert A, Lyantagaye SL, Nkwengulila G (2019). Farmers’ pesticide usage practices in the malaria endemic region of NorthWestern Tanzania: implications to the control of malaria vectors. BMC Public Health.

[9] Sharma A, Kumar V, Shahzad B, Tanveer M, Sidhu GPS, Handa N (2019). Worldwide pesticide usage and its impacts on ecosystem. SN Applied Sciences.

[10] Fianko JR, Donkor ST, Lowor PO, Yeboah ET, Glover T, Adom AF (2011). Health risk associated with pesticide contamination of fish from the Densu River Basin in Ghana. Journal of Environmental Protection.

[11] Olisah C, Okoh OO, Okoh AI (2020). Occurrence of organochlorine pesticide residues in biological and environmental matrices in Africa: A two-decade review. Heliyon.

[12] https://www.unep.org/topics/chemicals-and-pollution-action/pollution-and-health/persistent-organicpollutants-pops-4.

[13] Oyinloye JA, Oyekunle JAO, Ogunfowokan AO, Msagati T, Adekunle AS, Nety SS (2021). Human health risk assessments of organochlorine pesticides in some food crops from Esa-Oke farm settlement, Osun State, Nigeria. Heliyon.

[14] Ogbeide O, Tongo I, Enuneku A, Ogbomida E, Ezemonye L (2016). Human health risk associated with dietary and non-dietary intake of organochlorine pesticide residues from rice fields in Edo State Nigeria. Exposure and Health.

[15] Taiwo AM (2019). A review of environmental and health effects of organochlorine pesticide residues in Africa. Chemosphere.

[16] Sharma A, Shukla A, Attri K, Kumar M, Kumar P, Suttee A (2020). Global trends in pesticides: A looming threat and viable alternatives. Ecotoxicol Environ Saf.

[17] https://iris.epa.gov/AtoZ/?list_type=alpha.

[18] Infante-Rivard C, Weichenthal S (2007). Pesticides and childhood cancer: an update of Zahm and Ward's 1998 review. J Toxicol Environ Health B Crit Rev.

[19] Arrebola JP, Belhassen H, Artacho-Cordón F, Ghali R, Ghorbel H, Boussen H (2015). Risk of female breast cancer and serum concentrations of organochlorine pesticides and polychlorinated biphenyls: a case-control study in Tunisia. Sci Total Environ.

[20] Attaullah M, Yousuf MJ, Shaukat S, Anjum SI, Ansari MJ, Buneri ID (2018). Serum organochlorine pesticides residues and risk of cancer: A case-control study. Saudi J Biol Sci.

[21] Mazlan N, Ahmed M, Muharam FM, Alam MA (2017). Status of persistent organic pesticide residues in water and food and their effects on environment and farmers: a comprehensive review in Nigeria. Semina: Ciências Agrárias, Londrina.

[22] Akan JC, Jafiya L, Chellube ZM, Mohammed Z, Abdulrahman FI (2014). Determination of some organochlorine pesticide residues in vegetable and soil samples from Alau Dam and Gongulong Agricultural Sites, Borno State, North Eastern Nigeria. International Journal of Environmental and Ecological Engineering.

[23] Maurya PK, Malik DS (2016). Bioaccumulation of xenobiotics compound of pesticides in riverine system and its control technique: a critical review. Journal of Industrial Pollution Control.

[24] Adeyemi MM, Abdulmalik H (2017). Determination of organochlorine pesticides in some imported frozen fish species consumed within Kaduna Metropolis. IOSR Journal of Environmental Science, Toxicology and Food Technology (IOSRJESTFT).

[25] Osuala FI, Abiodun OA, Alebiosu EA (2020). Contamination levels of organochlorine pesticides in Tympanotonus fuscatus and sediment of Lagos Lagoon, Nigeria. Federal University Wukuri Trends in Science and Technology Journal.

[26] Oluwoyo T, Kocadal K, Saygi S, Battal D (2024). Determination of pesticide residue content in fruits and vegetables from Lagos, Nigeria. Environmental Analysis Health and Toxicology.

[27] Opeolu BO, Adenuga OO, Ndakidemi PA, Olujimi OO (2010). Assessment of phyto-toxicity potential of lead on tomato (Lycopersicon esculentum L) planted on contaminated soil. International Journal of Physical Sciences.

[28] https://nepis.epa.gov/Exe/ZyNET.exe/20014266.TXT?ZyActionD=ZyDocument&Client=EPA&Index=1995+Thru+1999&Docs=&Query=&Time=&EndTime=&SearchMethod=1&TocRestrict=n&Toc=&TocEntry=&QField=&QFieldYear=&QFieldMonth=&QFieldDay=&IntQFieldOp=0&ExtQFieldOp=0&XmlQuery=&File=D%3A%5Czyfiles%5CIndex%20Data%5C95thru99%5CTxt%5C00000019%5C20014266.txt&User=ANONYMOUS&Password=anonymous&SortMethod=h%7C-&MaximumDocuments=1&FuzzyDegree=0&ImageQuality=r75g8/r75g8/x150y150g16/i425&Display=hpfr&DefSeekPage=x&SearchBack=ZyActionL&Back=ZyActionS&BackDesc=Results%20page&MaximumPages=1&ZyEntry=1&SeekPage=x&ZyPURL.

[29] https://www.epa.gov/risk/regional-screening-levels-rsls-generic-tables.

[30] https://www.atsdr.cdc.gov/spl/index.html.

[31] Huang T, Guo Q, Tian H, Mao X, Ding Z, Zhang G (2014). Assessing spatial distribution, sources, and human health risk of organochlorine pesticide residues in the soils of arid and semiarid areas of northwest China. Environ Sci Pollut Res Int.

[32] Shah ZU, Parveen S (2023). Distribution and risk assessment of pesticide residues in sediment samples from river Ganga, India. PLoS One.

[33] Qu C, Qi S, Yang D, Huang H, Zhang J, Chen W (2015). Risk assessment and influence factors of organochlorine pesticides (OCPs) in agricultural soils of the hill region: A case study from Ningde, southeast China. Journal of Geochemical Exploration.

[34] Jayaraj R, Megha P, Sreedev P (2016). Organochlorine pesticides, their toxic effects on living organisms and their fate in the environment. Interdiscip Toxicol.

[35] Liu Y, Mo R, Tang F, Fu Y, Guo Y (2015). Influence of different formulations on chlorpyrifos behavior and risk assessment in bamboo forest of China. Environ Sci Pollut Res Int.

[36] Marie L, Sylvain P, Benoit G, Maurice M, Gwenaël I (2017). Degradation and transport of the chiral herbicide s-metolachlor at the catchment scale: Combining observation scales and analytical approaches. Environ Sci Technol.

[37] Pathak VM, Verma VK, Rawat BS, Kaur B, Babu N, Sharma A (2022). Current status of pesticide effects on environment, human health and it's eco-friendly management as bioremediation: A comprehensive review. Front Microbiol.

[38] Aiyesanmi AF, Idowu GA (2012). Organochlorine pesticides residues in soil of cocoa farms in Ondo State Central District, Nigeria. Environment and Natural Resources Research.

[39] Akinsanya B, Olaleru F, Samuel OB, Isibor PO, Adeniran OS (2021). Bioaccumulation of organochlorine pesticides, Procamallanus sp. (Baylis, 1923) infections, and microbial colonization in African snakehead fish sampled from Lekki Lagoon, Lagos, Nigeria. Braz J Biol.

[40] Sosan MB, Oyekunle JAO, Olufade YA (2015). Dichloro-diphenyl-trichloro-ethane (DDT) and hexachlorohexane (HCH) pesticide residues in foodstuffs from markets in Ile-Ife, Nigeria. International Journal of Biological and Chemical Sciences.

[41] Rajan S, Parween M, Raju NJ (2023). Pesticides in the hydrogeo-environment: a review of contaminant prevalence, source and mobilisation in India. Environ Geochem Health.

[42] https://www.atsdr.cdc.gov/ToxProfiles/tp12-p.pdf.

[43] https://iris.epa.gov/ChemicalLanding/&substance_nmbr=160.

[44] Gopalan NK, Chenicherry S (2018). Fate and distribution of organochlorine insecticides (OCIs) in Palakkad soil, India. Sustainable Environment Research.

[45] Tzanetou EN, Karasali H (2022). A comprehensive review of organochlorine pesticide monitoring in agricultural soils: The silent threat of a conventional agricultural past. Agriculture.

[46] Tariq SR, Shafiq M, Chotana GA (2016). Distribution of heavy metals in the soils associated with the commonly used pesticides in cotton fields. Scientifica(Cairo).

[47] Ibebuchi CC, Abu IO (2023). Rainfall variability patterns in Nigeria during the rainy season. Sci Rep.

[48] Nyantakyi JA, Wiafe S, Akoto O (2022). Seasonal changes in pesticide residues in water and sediments from River Tano, Ghana. J Environ Public Health.

[49] https://monographs.iarc.fr/wp-content/uploads/2018/09/List_of_Classifications.pdf.

[50] https://www.atsdr.cdc.gov/toxprofiles/tp1.pdf.

[51] https://www.atsdr.cdc.gov/toxprofiles/tp12.pdf.

[52] https://cfpub.epa.gov/si/si_public_record_report.cfm?Lab=NHSRC&dirEntryId=231764.

[53] Hossain MS, Fakhruddin ANM, Alamgir Zaman Chowdhury M, Rahman MA, Khorshed Alam M (2015). Health risk assessment of selected pesticide residues in locally produced vegetables of Bangladesh. International Food Research Journal.

[54] https://www.federalregister.gov/documents/1996/06/27/96-12991/minimal-risk-levels-for-priority-substances-and-guidance-for-derivation-republication.

[55] http://www.atsdr.cdc.gov/mrls.html.

[56] Yao S, Huang J, Zhou H, Cao C, Ai T, Xing H (2022). Levels, distribution and health risk assessment of organochlorine pesticides in agricultural soils from the Pearl River Delta of China. Int J Environ Res Public Health.

